# RNA origin of sex-biased immunity

**DOI:** 10.1016/j.omtn.2026.102853

**Published:** 2026-02-16

**Authors:** Howard Y. Chang, Lorinda Chung, Mark M. Davis, David Fiorentino, Jinwoo Lee, Emma Lundberg, P.J. Utz

**Affiliations:** 1 Department of Dermatology, Stanford University School of Medicine, Stanford, CA, USA; 2 Division of Immunology and Rheumatology, Department of Medicine, Stanford University School of Medicine, Stanford, CA, USA; 3 Institute of Immunity, Transplantation and Infection, and the Department of Microbiology and Immunology, Stanford University School of Medicine, Stanford, CA, USA; 4 Departments of Bioengineering and Pathology, Stanford University School of Medicine, Stanford, CA, USA; 5 RNA Medicine Program, Stanford University, Stanford, CA 94305, USA

## Main text

The human immune system exhibits a striking sex bias. Autoimmune diseases comprise a large set of disorders that together afflict 50 million Americans; four of five patients with autoimmunity are women. In systemic lupus erythematosus (SLE), the ratio of patient sex is 9:1 females to males; the ratio in Sjögren’s disease is 19:1 female to male patients.^1^ Why are the immune systems of men and women different? Conventional explanations invoke differences in sex hormones or sex chromosomes. Women have two X chromosomes (XX) and men have one X and one Y (XY) chromosome, with the Y-linked genes driving male characteristics. Importantly, patients with Klinefelter syndrome (XXY) are phenotypically male, have male hormonal patterns, but have an elevated risk of autoimmune disease equivalent to females.^1^ This observation suggests that it is not the sex hormones but the presence of the second X chromosome or the machinery associated with the second X that may be responsible. Recently, our team made the surprising discovery that a single long noncoding RNA (lncRNA) named Xist and its protein partners are the major drivers for female-biased immunity.^2^ A male mouse engineered to express Xist results in female-level autoimmune disease.^2^ This discovery fundamentally changes our concept of sex-biased immunity. Here, we outline the origins of this hypothesis, experiments to evaluate this idea, and translational opportunities to understanding how Xist ribonucleoproteins (RNPs) might drive autoimmunity.

To make the gene expression output roughly equivalent between females and males, every cell in a female mammal’s body epigenetically silences one of two X chromosomes via the action of the lncRNA, Xist. Xist is a ∼17-kb lncRNA that is transcribed only from the inactive X chromosome and thus not expressed in males. Xist is critical for the establishment of X chromosome inactivation (XCI), spreading from its site of transcription, coating the entire inactive X chromosome to form the condensed Barr body.^3^ Xist is also expressed in adult somatic cells and required to maintain the silencing of a subset of X-linked genes, including the toll-like receptor 7 (*TLR7*) gene in B cells.^4^ Xist associates with 81 proteins to form a RNP complex during XCI establishment in embryonic stem cells and an additional set of cell-type specific RNP subunits in adult somatic cells.^3^^,^^4^ These proteins are present in male cells but are not assembled into a unique RNP in the absence of Xist. Then a serendipitous discovery changed the course of our research: as a dermatologist-scientist, one of us (H.Y.C.) was studying for a medical certification renewal and memorizing lists of autoantigens. H.Y.C. noticed that nearly a quarter of top Xist-enriched proteins (being studied in his laboratory) are known autoantigens, i.e., the targets of autoantibodies in patients with autoimmune disease including SLE, scleroderma, Sjögren’s disease, and rheumatoid arthritis. Physicians and scientists have long noted that many autoantibodies target large nucleic acid-protein complexes, such as chromatin or RNP, in human autoimmune diseases. Specifically, these immune targets include components of the centromere complex (systemic sclerosis), the spliceosome (SLE), and nucleic acid complexes that regulate transcription and chromatin accessibility (dermatomyositis). Immunologists have explained this phenomenon with the idea that large nucleic acid-protein complexes are polymeric and can cluster and/or assemble in alternative liquid-liquid phase complexes that, if exposed in the extracellular space, can activate immunoreceptors. We proposed that the XIST RNP is one such dominant antigenic complex that is unique to females. Every cell in a woman’s body has XIST, which is a long polymer and coats the entire inactive X chromosome as the Barr body (an even larger polymer). When a female cell dies due to tissue injury, XIST RNPs will invariably be exposed to the immune system, which may contribute to several steps in the progression to autoimmune disease.

A rigorous test of the Xist RNP hypothesis is to express Xist in male animals. If Xist expression in males sufficed to confer female-level autoimmunity, it would mean that neither female sex hormones nor double X chromosome dosage is required. Such a model will allow one to investigate the singular contribution of Xist RNP to sex-biased immunity. To this end, our team developed a TetOP-**Δ**RepA-Xist transgenic mouse that enables inducible expression of *Xist* in male animals. Because Xist expression from an autosome silences the chromosome in *cis* and is often cell lethal, we chose to use **Δ**RepA-Xist, a truncation of Xist that removes the A-repeat (RepA) element required for gene silencing activity of Xist but does not ablate chromosome coating or Xist RNP formation.^5^ We previously found that 78 of 81 proteins in the Xist RNP associate with **Δ**RepA-Xist.^3^ After only 2 weeks of doxycycline administration in tgXist male mice, expression of tgXist was induced ∼100-fold in multiple tissues to approximately the level of endogenous Xist in females; RNA fluorescence *in situ* hybridization revealed tgXist localized as single punctate foci in the nucleus reminiscent of Barr body.

The pristane-induced SLE mouse model exhibits many characteristics of human SLE and importantly demonstrates a strong female bias.^6^ Female mice display earlier mortality, more severe nephritis, higher levels of autoantibodies, and are 3.4-fold more likely to die than male mice. Female WT mice demonstrated multiorgan disease in adipose tissues, lymph nodes, kidney, liver, and lung. Male WT mice were comparatively resistant, while 5 of 8 t*gXist* male mice exhibited multi-organ pathology on par with female mice. Both pristane and Xist induction were required for this effect. Single-cell RNA sequencing analysis of our mouse cohorts revealed that autoimmune disease induction in affected tgXist male mice and wild-type female mice was most prominently associated with expansion of atypical B cells (ABCs). ABCs are noncanonical memory B cells that often have autoreactive specificities and are known to accumulate preferentially in aged female mice.^4^ The ability of Xist to induce ABCs *in vivo* unifies these two areas of research and suggests potential cellular mediators of Xist-driven autoimmunity.

To test whether the XIST RNP is immunogenic in humans, we created a protein array of Xist RNP subunits, composed of 128 Xist-associated proteins and 52 control proteins. Profiling sera from 108 patients with SLE, systemic sclerosis, and dermatomyositis and 16 age matched healthy controls revealed that sera from the 3 disease groups were significantly reactive to 53 unique proteins in the Xist RNP compared to healthy controls. The pattern of Xist RNP autoantibodies distinguished the three diseases, and 28 proteins in the Xist RNP are nominated as autoantigens for the first time. These results suggest that the Xist RNP is a major target of autoantibodies in human female-biased autoimmunity, with diagnostic potential.

This work has led to the establishment of a Specialized Center of Research Excellence on Sex Differences in Autoimmunity (SCORE-X) at Stanford University. SCORE-X aims to advance sex-biased autoimmunity in an interdisciplinary manner. Human organoids and spatial proteomics will be employed to dissect the role of Xist RNP in driving autoimmunity, focusing on the diagnostic and prognostic opportunities from newly discovered anti-XIST RNP antibodies. We hope that SCORE-X will enhance collaborative, research, and educational opportunities to benefit patients living with autoimmune diseases.

## Declaration of interests

H.Y.C. is an employee and stockholder of Amgen as of December 16, 2024. H.Y.C. is a co-founder of Accent Therapeutics, Boundless Bio, Cartography Biosciences, and Orbital Therapeutics. H.Y.C was an advisor of 10× Genomics, Arsenal Bio, Chroma Medicine, and Exai Bio until December 15, 2024. M.M.D. is a scientific co-founder, member of the Scientific Advisory Board, paid consultant, and equity holder of Mozart Therapeutics, Inc. E.L. is an advisor for the Chan-Zuckerberg Initiative Foundation, Element Biosciences, Cartography Biosciences, Pfizer, GenBio.AI, and Pixelgen Technologies AB. P.J.U. serves on the scientific advisory boards of SeraNova, 4DMT, Yolo Therapeutics, and the Arthritis National Research Foundation. He is co-founder and member of the Board of Directors of the Physician Scientist Support Foundation.
